# Author Correction: A critical period of translational control during brain development at codon resolution

**DOI:** 10.1038/s41594-025-01675-6

**Published:** 2025-08-27

**Authors:** Dermot Harnett, Mateusz C. Ambrozkiewicz, Ulrike Zinnall, Alexandra Rusanova, Ekaterina Borisova, Amelie N. Drescher, Marta Couce-Iglesias, Gabriel Villamil, Rike Dannenberg, Koshi Imami, Agnieszka Münster-Wandowski, Beatrix Fauler, Thorsten Mielke, Matthias Selbach, Markus Landthaler, Christian M. T. Spahn, Victor Tarabykin, Uwe Ohler, Matthew L. Kraushar

**Affiliations:** 1 Berlin Institute for Medical Systems Biology, Max Delbrück Center for Molecular Medicine, Berlin, Germany; 2 Institute of Cell Biology and Neurobiology, Charité-Universitätsmedizin Berlin, corporate member of Freie Universität Berlin, Humboldt-Universität zu Berlin, and Berlin Institute of Health, Berlin, Germany; 3 Institute of Neuroscience, Lobachevsky University of Nizhny Novgorod, Nizhny Novgorod, Russian Federation; 4 Max Planck Institute for Molecular Genetics, Berlin, Germany; 5 RIKEN Center for Integrative Medical Sciences, Yokohama, Japan; 6 Max Delbrück Center for Molecular Medicine, Berlin, Germany; 7 Institute of Neuroanatomy, Charité-Universitätsmedizin Berlin, Berlin, Germany; 8 Institute of Biology, Humboldt University of Berlin, Berlin, Germany; 9 Institute of Medical Physics and Biophysics, Charité-Universitätsmedizin Berlin, Berlin, Germany; 10 Department of Computer Science, Humboldt University of Berlin, Berlin, Germany

**Keywords:** Ribosome, Systems biology, Stem cells

Correction to: *Nature Structural & Molecular Biology* 10.1038/s41594-022-00882-9, published online 8 December 2022.

In the version of this article initially published online, the Gene Ontology pathways listed in Fig. 7b were incorrect due to a plotting error, while the sentence at the end of the first paragraph of the “Modeling the translatome of neocortex neurogenesis” section, now reading, in part, “Reinforcing the biological significance of different mRNA and protein trajectories… neuron differentiation processes enriched in cluster G,” “cluster G” originally read “cluster D.” The figure and text have been updated in the HTML and PDF versions of the article. For comparison, the original Fig. 7 is available as Supplementary Information alongside this amendment.

## Supplementary information

Original, uncorrected Fig. 7

