# Supplementary material for: A critical period of translational control during brain development at codon resolution
Source: Nat Struct Mol Biol. 2022 Dec 8;29(12):1277–90. doi: 10.1038/s41594-022-00882-9 (PMC9758057; doi:10.1038/s41594-022-00882-9)
Supplement: Supplementary file 1 — Reporting Summary [file 41594_2022_882_MOESM1_ESM.pdf]

Corresponding author(s): Matthew L. Kraushar
Dermot Harnett
Mateusz C. Ambrozkiewicz

Last updated by author(s): Sep 6, 2022

Reporting Summary

Nature Portfolio wishes to improve the reproducibility of the work that we publish. This form provides structure for consistency and transparency in reporting. For further information on Nature Portfolio policies, see our Editorial Policies and the Editorial Policy Checklist.

Statistics

For all statistical analyses, confirm that the following items are present in the figure legend, table legend, main text, or Methods section.

n/a	Confirmed	
☐	☒	The exact sample size (*n*) for each experimental group/condition, given as a discrete number and unit of measurement
☐	☒	A statement on whether measurements were taken from distinct samples or whether the same sample was measured repeatedly
☐	☒	The statistical test(s) used AND whether they are one- or two-sided<br>*Only common tests should be described solely by name; describe more complex techniques in the Methods section.*
☐	☒	A description of all covariates tested
☐	☒	A description of any assumptions or corrections, such as tests of normality and adjustment for multiple comparisons
☐	☒	A full description of the statistical parameters including central tendency (e.g. means) or other basic estimates (e.g. regression coefficient) AND variation (e.g. standard deviation) or associated estimates of uncertainty (e.g. confidence intervals)
☐	☒	For null hypothesis testing, the test statistic (e.g. *F*, *t*, *r*) with confidence intervals, effect sizes, degrees of freedom and *P* value noted<br>*Give P values as exact values whenever suitable.*
☐	☒	For Bayesian analysis, information on the choice of priors and Markov chain Monte Carlo settings
☐	☒	For hierarchical and complex designs, identification of the appropriate level for tests and full reporting of outcomes
☐	☒	Estimates of effect sizes (e.g. Cohen's *d*, Pearson's *r*), indicating how they were calculated

Our web collection on statistics for biologists contains articles on many of the points above.

Software and code

Policy information about availability of computer code

Data collection	No commercial, open source, or custom code was utilized for data collection.
Data analysis	For data analysis, code generated during this study is supplied at: https://github.com/ohlerlab/cortexomics. Further requests may be directed to and will be fulfilled by the Lead Contact, matthew.kraushar@molgen.mpg.de (M.L.K.). Code or software packages derived from previously published work are described with referenced sources in the Methods, which includes the following: Bcl2fastq 2.20.0, STAR 2.6.1a, Limma, Xtail 1.1.5, ProDA, RUST, DeepShapePrime, Salmon 0.14.1, Snakemake, Tximport, Voom, RiboDiPA, TopGO, MaxQuant v1.6.0.1, NanoString, Hclust, AME/Meme suite 5.1.1, Pqsfinder 2.10.1, Cutadapt 1.18, Graph Pad Prism 7, FIJI, IBAQ, LFQ, Perseus, R v4.0.0, SPSS v.17, Ribo-seQC

For manuscripts utilizing custom algorithms or software that are central to the research but not yet described in published literature, software must be made available to editors and reviewers. We strongly encourage code deposition in a community repository (e.g. GitHub). See the Nature Portfolio guidelines for submitting code & software for further information.

Data

Policy information about availability of data

All manuscripts must include a data availability statement. This statement should provide the following information, where applicable:

- Accession codes, unique identifiers, or web links for publicly available datasets
- A description of any restrictions on data availability
- For clinical datasets or third party data, please ensure that the statement adheres to our policy

Data have been deposited in publicly available repositories as indicated:
RNA-seq data are publicly available in the NIH GEO: GSE157425.
Ribo-seq data are deposited in the NIH GEO: GSE169457.

tRNA qPCR array data are deposited in the NIH GEO: GSE169621.
Mass spectrometry data are publicly available in the ProteomeXchange: PXD014841.
This study further utilized GENCODE release M12.

Field-specific reporting

Please select the one below that is the best fit for your research. If you are not sure, read the appropriate sections before making your selection.

☒ Life sciences ☐ Behavioural & social sciences ☐ Ecological, evolutionary & environmental sciences

For a reference copy of the document with all sections, see nature.com/documents/nr-reporting-summary-flat.pdf

Life sciences study design

All studies must disclose on these points even when the disclosure is negative.

Sample size

No sample-size calculation was performed. All replicates reported constitute biological replicates. The sample-size for biological replicates was determined based on standards in the fields of RNA sequencing & Ribosome Profiling (Ingolia et al. Science. (324) 2009), tRNA analysis (Zheng et al. Nat Methods. (12) 2015), and mass spectrometry (Tabb et al. J Proteome Res. (9) 2010), which includes the extent of practicable feasibility and financial limitations of replicate measurements. As described in detail in the Methods, this includes biological replicates n = 2 for RNA-seq, Ribo-seq, tRNA qPCR array; n = 3 for mass spectrometry. All biological replicates and sample-sizes for quantification of fluorescent in situ hybridization, immunohistochemistry, and in utero electroporation constitute n ≥ 3, and were determined in part by the availability and financial cost of replicate animals and reagents - all such n's are detailed in the Methods and Supplementary Table 3.

Data exclusions

No data were excluded from analyses.

Replication

Reproducibility for all experiments were assessed statistically. Consistency of our findings, including significant differences between conditions, were tested with the reported statistical tests in the Methods, Figure Legends, and Supplementary Tables. All numerical values and description of statistical tests used, definition of center, dispersion, precision, and definition of significance can be found in the Supplementary Tables and in the Methods. Prior to comparison of experimental groups, normality and log-normality test were performed. Reproducibility of non-quantitative data, such as fluorescence or electron microscopy images, were confirmed across multiple replicate images derived from independent animals and electroporations.

Randomization

Mice were utilized in the embryonic (E12.5-E17) and early post-natal (P0) period, inclusive of both male and female sexes in each litter without distinction. Sequencing (n = 2), qPCR-array (n = 2), and mass spectrometry (n = 3) experiments pooled multiple animals' cortex tissue across multiple independent litters for each biological replicate. The assignment of wild type animals into omics, FISH, and IHC experimental groups was not randomized, but simply followed their developmental stage. For in utero electroporation experiments, animals within the same litter either received control or overexpression plasmids; thus while not randomized, the proper intervention-control comparisons were performed appropriate for statistical comparison.

Blinding

Investigators were blinded for both imaging and quantification of immunohistochemistry, in situ hybridization, and in utero electroporation experiments, as the investigators themselves were responsible for deriving quantitative data from microscopy image data. For computational bioinformatic analysis of sequencing, qPCR array, and mass spectrometry data, investigators were not blinded to sample condition. We justify the bioinformatics being un-blinded because the computational algorithms/scripts were written in advance of the analysis and quantification, and were run exactly the same between all experimental groups.

Reporting for specific materials, systems and methods

We require information from authors about some types of materials, experimental systems and methods used in many studies. Here, indicate whether each material, system or method listed is relevant to your study. If you are not sure if a list item applies to your research, read the appropriate section before selecting a response.

Materials & experimental systems

n/a	Involved in the study	
☐	☒	Antibodies
☒	☐	Eukaryotic cell lines
☒	☐	Palaeontology and archaeology
☐	☒	Animals and other organisms
☒	☐	Human research participants
☒	☐	Clinical data
☒	☐	Dual use research of concern

Methods

n/a	Involved in the study	
☒	☐	ChIP-seq
☒	☐	Flow cytometry
☒	☐	MRI-based neuroimaging

Antibodies

Antibodies used

As described in the Methods, primary antibodies used for immunocytochemistry were used at dilutions indicated: anti-Satb2 (1:500, rabbit, home-made; Ambrozkiewicz et al. J Neurosci Methods. (291) 2017), anti-Bcl11b/Ctip2 (1:500, rat, Abcam, 25B6,

RRID:AB_2064130), anti-GFP (1:1000, goat, Rockland, RRID:AB_2612804), anti-Cre (1:1000, rabbit, SySy, RRID:AB_2619968), anti-Tbr2 (1:300, rabbit, Abcam, RRID:AB_778267), anti-Pax6 (1:500, rabbit, Millipore, RRID:AB_1587367), Draq5 (1:2000), anti-eIF4EBP1 (1:1000, rabbit, Abcam, ab32024, RRID:AB_2097990), anti-phospho-eIF4EBP1 Thr37/46 (1:1000, rabbit, Cell Signaling, 2855, RRID:AB_560835), anti-Rps5 a.k.a. uS7 (1:1000, mouse, Santa Cruz, sc-390935, RRID:AB_2713966). All secondary Cy2, Cy3, and Cy5 antibodies were from Jackson Immunoresearch and were used at 1:250.

Primary antibodies used for Western blot were used at the dilutions indicated: anti-eIF4EBP1 (1:1000, rabbit, Abcam, ab32024, RRID:AB_2097990), anti-phospho-eIF4EBP1 Thr37/46 (1:1000, rabbit, Cell Signaling, 2855, RRID:AB_560835), anti-Gapdh (1:1000, mouse, Millipore, MAB374, RRID:AB_2107445). All Horse Radish Peroxidase secondary antibodies were used at 1:2500: HRP-anti-mouse-Heavy Chain (goat, Abcam, ab97245; RRID:AB_10680049), HRP-anti-rabbit-Heavy Chain (goat, Cell Signaling, 7074S, RRID:AB_2099233).

Validation

The antibody anti-Satb2 (rabbit, home-made; Ambrozkiewicz et al. J Neurosci Methods. (291) 2017) was validated by immunohistochemistry in Satb2 -/- (knockout) brains as described in the Extended Data. The antibody anti-Rps5 a.k.a. uS7 (mouse, Santa Cruz, sc-390935, RRID:AB_2713966) was further validated by western blot probing of total neocortex lysates across all developmental stages (E12.5-P0) in a prior study from the authors (Kraushar et al. Mol Cell. (81) 2021), demonstrating a clear single band at the appropriate molecular weight. Otherwise, we did not independently validate the other antibodies used; beyond what is annotated manufacturers' standard methods, which include western blot testing of genomic knock-out lysates, and/or in comparison to recombinantly expressed protein corresponding to the immunogen. We defer to the manufacturer-specific details, which are searchable at the catalogue IDs and RRIDs provided for all antibodies used in our study.

Animals and other organisms

Policy information about studies involving animals; ARRIVE guidelines recommended for reporting animal research

Laboratory animals

Mice were housed in 12 hour light/12 hour dark cycle, at a consistent 18-23 °C, 40-60% humidity, with pellet food and water available ad libitum. Mice were utilized in the embryonic (E12.5-E17) and early post-natal (P0) period with the stage and replicate numbers as reported for each experiment. Each sample was inclusive of both male and female sexes in each litter without distinction. Timed pregnant wild-type (WT) CD-1 mice utilized for Ribo-seq, RNA-seq, tRNA qPCR array, mass spectrometry, and immuno-electron microscopy were obtained from the Charles River Company (Protocol: T0267/15). Experiments with fluorescent in situ hybridization and immunohistochemistry were performed in NMRI WT mice. For experiments with the tdTomato reporter, Satb2Cre/+ males (Britanova et al. Neuron. (57) 2008) were mated to NMRI wild type females (Protocols: G0079/11, G54/19, and G206/16). Satb2Cre/+ mouse genotyping was performed as described (Britanova et al. Neuron. (57) 2008).

Wild animals

The study did not involve wild animals.

Field-collected samples

The study did not involve animals collected from the field.

Ethics oversight

Mouse (Mus musculus) lines were maintained in the animal facilities of the Charité University Hospital and Lobachevsky State University. All experiments were performed in compliance with the guidelines for the welfare of experimental animals approved by the State Office for Health and Social Affairs, Council in Berlin, Landesamt für Gesundheit und Soziales (LaGeSo), permissions T0267/15, G0079/11, G206/16, and G54/19, and by the Ethical Committee of the Lobachevsky State University of Nizhny Novgorod.

Note that full information on the approval of the study protocol must also be provided in the manuscript.
