# Supplementary material for: A critical period of translational control during brain development at codon resolution
Source: Nat Struct Mol Biol. 2022 Dec 8;29(12):1277–90. doi: 10.1038/s41594-022-00882-9 (PMC9758057; doi:10.1038/s41594-022-00882-9)
Supplement: Supplementary file 8 — Full unprocessed western blots for Fig. 6. [file 41594_2022_882_MOESM8_ESM.pdf]

Source Data Files

Fig. 6d. Full Western blots

Each stage probed in biological duplicate (two lanes), comprising multiple brain lysates per lane.
Exact same samples/amounts loaded between blots
Ladder for all is SeeBlue Plus2 (Invitrogen LC5925)

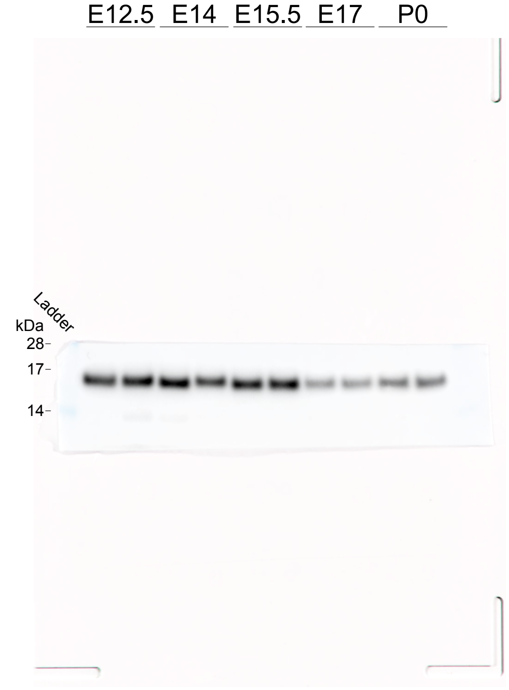

anti-eIF4EBP1

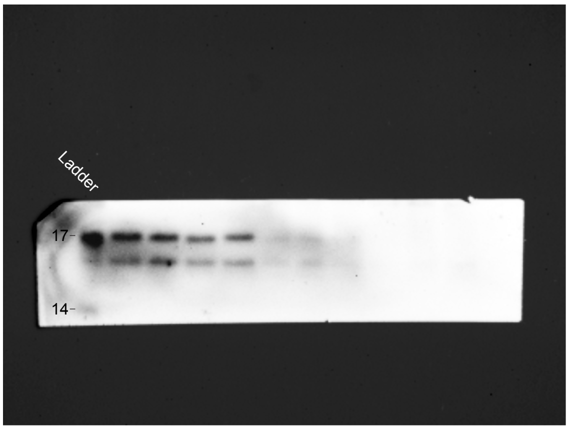

anti-p-eIF4EBP1

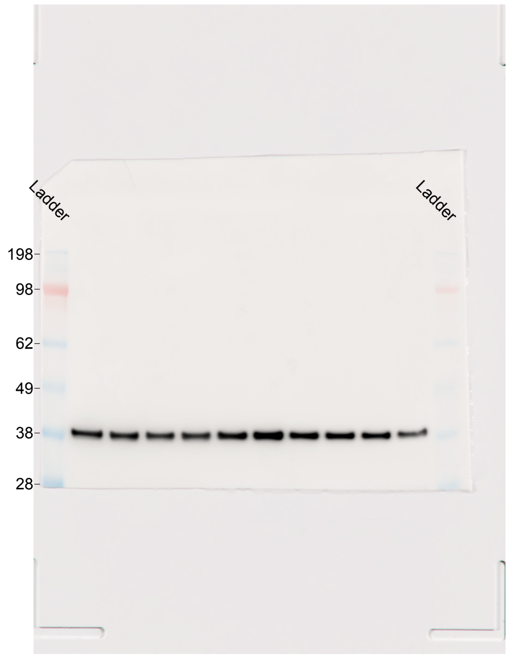

anti-Gapdh
