# Supplementary material for: Author Correction: A critical period of translational control during brain development at codon resolution
Source: Nat Struct Mol Biol. 2025 Aug 27;32(9):1838. doi: 10.1038/s41594-025-01675-6 (PMC12440796; doi:10.1038/s41594-025-01675-6)

nature structural & molecular biology

Supplementary information

Author Correction: A critical period of translational control during brain development at codon resolution

In the format provided by the authors and unedited

Figure 7: Original

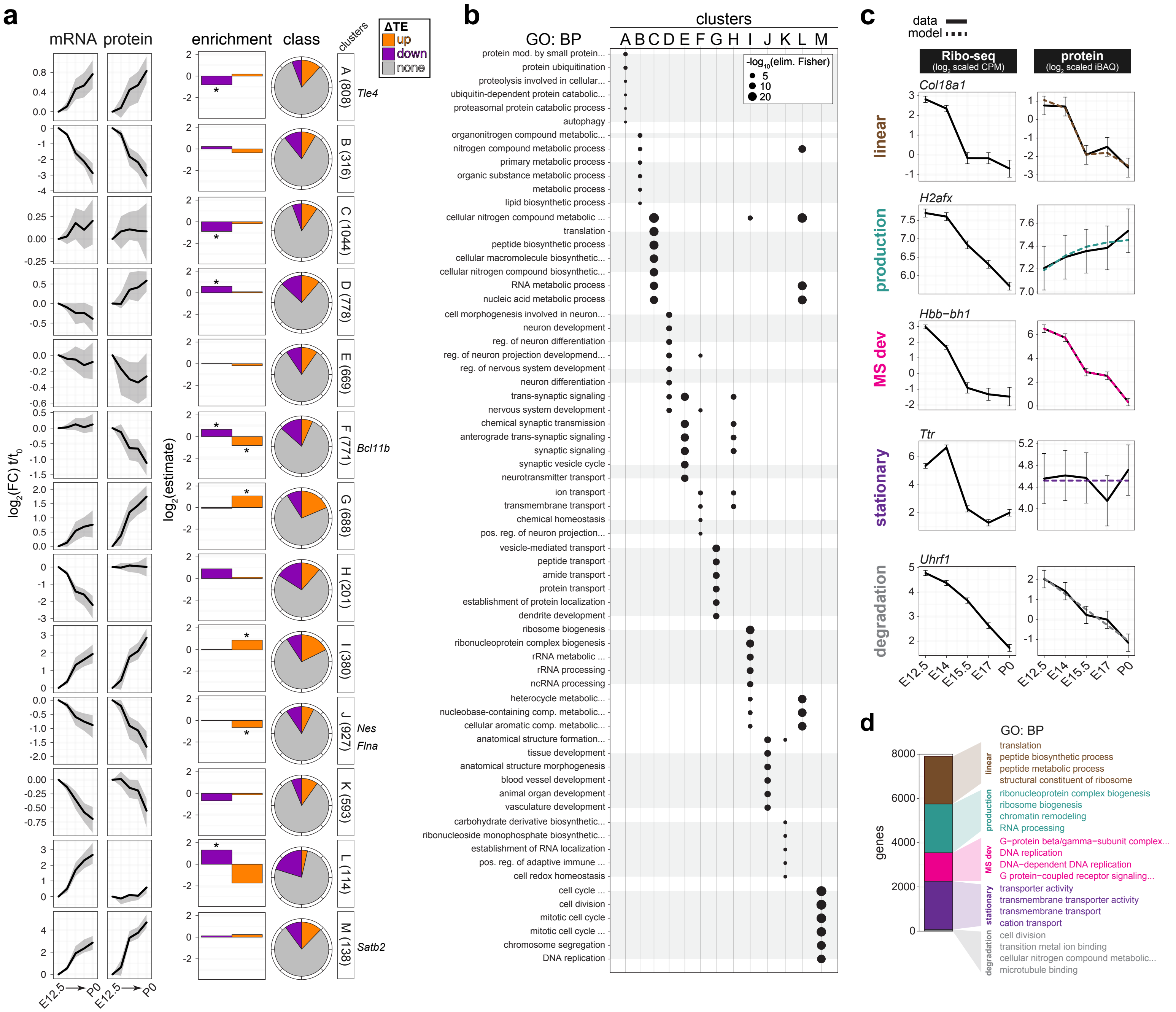

Supplement: Supplementary file 1 — Original, uncorrected Fig. 7 [file 41594_2025_1675_MOESM1_ESM.pdf]
